# Orbital Reef and commercial low Earth orbit destinations—upcoming space research opportunities

**DOI:** 10.1038/s41526-024-00363-x

**Published:** 2024-03-29

**Authors:** Luis Zea, Liz Warren, Tara Ruttley, Todd Mosher, Laura Kelsey, Erika Wagner

**Affiliations:** 1 Sierra Space, Broomfield, CO 80021 USA; 2 Blue Origin, Kent, WA 98032 USA

**Keywords:** Technology, Funding, Industry

## Abstract

As the International Space Station comes to the end of a transformative era of in-space research, NASA’s Commercial Low Earth Orbit (LEO) Destinations (CLD) Program aims to catalyze a new generation of platforms with co-investment from the private sector, preventing a potential gap in research performed in LEO, while building a robust LEO economy. In this paper, we provide insight into the CLD Program focusing on Orbital Reef, describing its operational and technical characteristics as well as new opportunities it may enable. Achieving about a third of the pressurized volume of the ISS with the launch of a single pressurized module and growing to support hundreds of Middeck Locker Equivalents (MLE) in passive and active payloads internally and externally, Orbital Reef will enable government, academic, and commercial institutions to continue and expand upon research and development (R&D) efforts currently performed on ISS. Additionally, it will enable nascent markets to establish their operations in space, by initiating new lines of research and technology development and the implementation of new ventures and visions. Using Blue Origin’s New Glenn heavy launch system, Sierra Space’s cargo and crew Dream Chaser® vehicles, and Boeing’s Starliner crew vehicle, and expertise from Amazon/Amazon Supply Chain, Arizona State University, Genesis Engineering, and Redwire, Orbital Reef is being designed to address ISS-era transportation logistics challenges. Finally, this manuscript describes some of the expected challenges from the ISS-to-CLD transition, and provides guidance on how researchers in academia and industry can shape the future of commercial destinations and work performed in LEO.

## Introduction

The International Space Station (ISS) is a multi-nation project, the single largest structure ever put in Earth’s orbit. It has hosted over 250 people from over 20 countries and over 3000 completed research investigations since assembly began in 1998^1^. Several of these efforts have translated into scientific and technical breakthroughs and have enabled advancements in a myriad of fields. For example, protein crystal growth investigations performed on ISS have provided novel insights into multiple disease treatments, as have fundamental and applied research on Alzheimer’s Disease, Parkinson’s Disease, heart disease, and various cancers^2^. The ISS has opened new fields of inquiry and fostered innovative techniques and capabilities, including the use of tissue chips in space, quiescent colloid research, 3D printing in microgravity, the development of new water purification systems, and lessons in how to live and thrive in space for longer durations^2^. As the ISS comes to the end of its design life, NASA now plans to retire the platform in 2030, and has established the Commercial LEO Destinations (CLD) Program to plan for the future^3,4^.

### NASA’s commercial LEO destinations (CLD) program

NASA’s commercial LEO programs are designed to support a transition to commercially owned and operated services that support the agency’s science, technology maturation, and astronaut training needs in Earth orbit^3,5,6^. In early 2020, NASA awarded a contract for commercial modules to be attached to the ISS to Axiom Space^3,7^. The Commercial Destinations-Free Flyer program was established in 2021 as a two-phase acquisition. In December 2021, NASA announced three awards through 2025: Northrop Grumman, Nanoracks “Starlab”, and Orbital Reef^5^. In Phase 1, private industry, in coordination with NASA, designs CLD capabilities to address government and commercial needs. Phase 2 will support certification of one or more platforms for U.S. government astronauts and end-to-end services including ground support, transportation, and on-orbit operations^5,6^. These services will ensure NASA’s ability to maintain a permanent presence in LEO with at least two crew members, performing approximately 200 scientific investigations annually^5^. These services are intended to be operational in the late 2020s to prevent a gap in LEO presence for the U.S. and its partners, and to ensure a smooth transition of operations from ISS^3,6^.

### A self-reinforcing ecosystem in space: an Orbital Reef

Orbital Reef will be a mixed-use business and research park; one of the world’s first commercial free-flying space stations. It is being jointly developed across the U.S. by Blue Origin, with Sierra Space, Boeing, Redwire Space, Amazon/Amazon Supply Chain, Genesis Engineering, and Arizona State University as teammates. Orbital Reef’s name follows the premise under which it is being developed: to serve as a robust infrastructure for a diverse, growing, and self-reinforcing ecosystem in Earth’s orbit, much like the ecosystems of coral reefs in the ocean. By providing a turnkey service with accessible infrastructure in LEO, Orbital Reef lowers the barriers to entry, and enables existing and nascent users to establish their operations in space. Customized spaces within Orbital Reef allow for government and private institutions, as well as commercial and academic entities to implement new ventures and visions, ranging from basic and applied R&D to entertainment and hospitality to port-of-call for exploration missions^8^. The station will be occupied by a mix of professional Orbital Reef crew members, national astronauts, and commercial users. Orbital Reef will have the capabilities needed to address scientific research and technology maturation currently being performed on ISS, ensuring the smooth transition envisioned by NASA’s CLD Program.

**Fig. 1:**
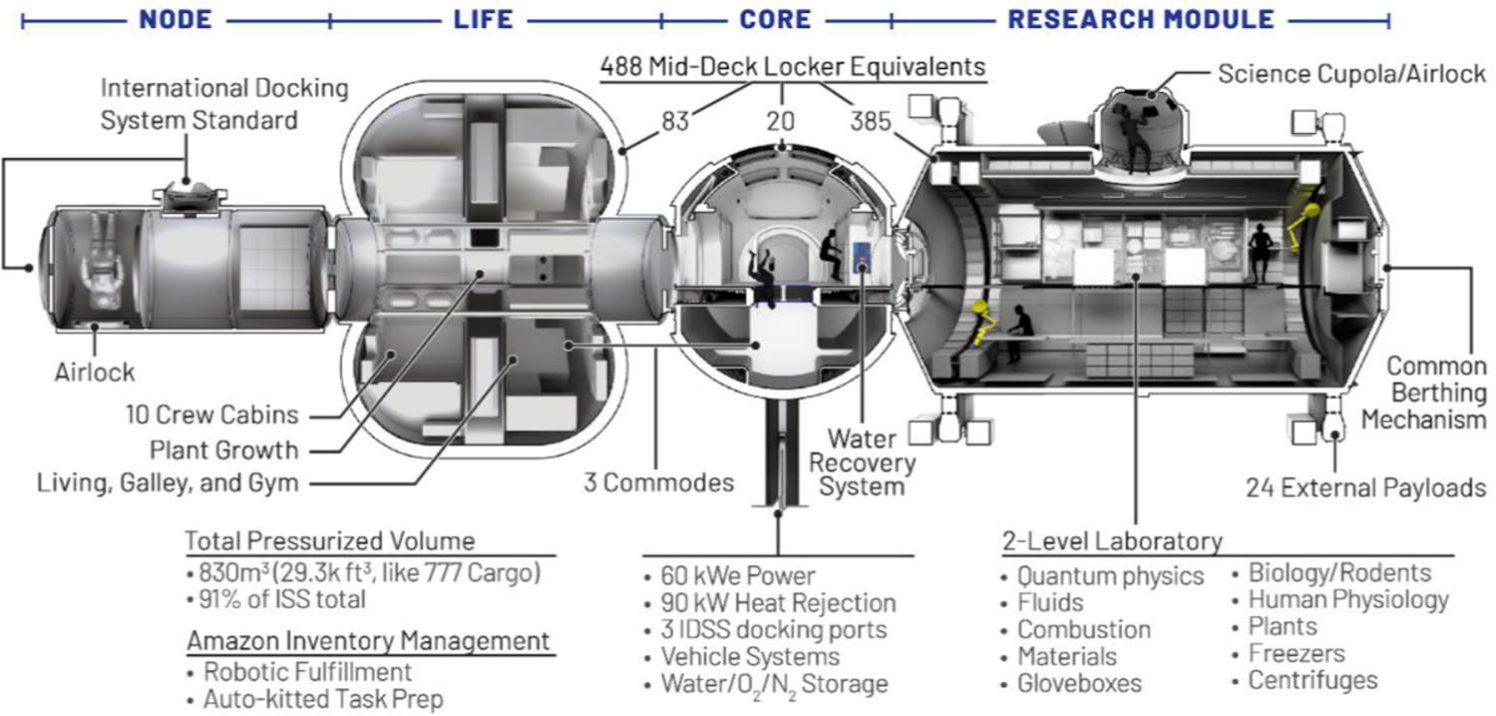
Orbital Reef’s modular pressurized elements: Node, LIFE^TM^, Core, and Research Module from Mosher & Kelsey, 2023.

In its initial phase, Orbital Reef will consist of (i) a Node and (ii) a Large Integrated Flexible Environment (LIFE^TM^) developed by Sierra Space, (iii) a Core module and (iv) a power/thermal Mast developed by Blue Origin, and (v) a Research Module developed by Boeing (Fig. 1). The Core, with 250 m^3^ of habitable volume, nearly a third of that available on the ISS, will serve as the central hub for the rest of Orbital Reef’s modules and visiting vehicles, as well as the center for command and control, data processing, and communications with the ground. It will host internal and external payloads, stowage, an environmental control and life support system (ECLSS) sized to support up to ten crewmembers, a commode, and will include six of the largest windows ever flown to space facing Earth. The Research Module, similar in size to the Core, will include a payload airlock/cupola and will host internal and external payloads, serving as a multi-disciplinary laboratory, customizable to user requirements. The Mast will generate 100 kWe through its deployable solar arrays. It will collect and reject heat, and will serve as the bus for communications and other critical systems, including an external robotic arm and docking and berthing nodes. The LIFE™ habitat is an expandable module that will provide over 300 m^3^ of habitable volume to host payloads and research facilities, an ECLSS and sleeping quarters for up to ten crewmembers, two commodes, a health and hygiene compartment, a galley, exercise equipment, and plant growth hardware. Finally, the ~40 m^3^ Node will include two International Docking System Standard (IDSS)-compatible visiting vehicle ports, an airlock for extravehicular activity (EVA), and will be able to host external payloads and provide station-keeping functions. More details on each of these modules and their operations are described in Mosher & Kelsey, 2023.

This modular architecture can be scaled through linear addition of additional Core and Mast modules, which can each support additional tenant modules to support growing market demand and new functionality.

## Upcoming opportunities

### Capabilities

Across its modules, Orbital Reef will have hundreds of MLE worth of volume to host passive and active payloads in addition to state-of-the-science research facilities to enable the continuation and expansion of R&D currently performed on ISS, as well as to initiate new lines of research and technology development. To achieve a smooth transition from ISS, Orbital Reef’s payload interfaces will be backwards-compatible with ISS MLE standards, i.e., hardware used on the ISS will be by default compatible. Additionally, Orbital Reef’s interfaces will offer optional upgraded interfaces to optimize processes and crew time. Orbital Reef will have the capability to host external payloads, accessible through a science airlock and external robotics, which will transfer them from visiting vehicles. An airlock will provide external access to astronauts via Extravehicular Activity (EVA). Additionally, co-orbiting free-flyers from any customer may be qualified for deployment from Orbital Reef or visiting vehicles to serve as platforms for autonomous exploration experiments, isolated microgravity environments, and unique views of the Orbital Reef, the Earth, and deep space. Similarly, full modules developed by third parties may be attached to Orbital Reef, receiving utilities (power, life support, etc.) and services to enable focus on their particular use cases.

For internal payloads, Orbital Reef will provide power at 28 VDC, 120 VDC, and 120 VAC; data transmission via 10 G Base-T ethernet and Wi-Fi; 36 kW of heat rejection; and nitrogen, carbon dioxide, water, air, and vacuum exhaust distribution hardware. Orbital Reef crew time can be made available for the operation of customer payloads and payload facilities, photography and videography, and other activities. Conversely or in parallel, customers themselves may work and live on Orbital Reef with the opportunity to bring their own payload facilities, as needed. Payload facilities will provide the capabilities needed to address multiple scientific and technical R&D and use cases. These include freezer banks (−80 °C and -20 °C), refrigeration (4 °C), incubators, separate life and physical sciences gloveboxes, microscopes, optics bench platform, 3-D printers, biofabrication banks, production facilities, a pressurized gas tank farm, and areas designated for NASA and commercial payload facilities and devices or for multi-purpose use. Externally, Orbital Reef will provide powered payloads with up to 2 kW of power distributed at 120 VDC and 10 G Base-T ethernet. Mounts will be compatible with small, medium, and large on-orbit replaceable unit (ORU) robotics interface standards (SORI, MORI, and LORI, respectively).

### Designed to solve ISS-era challenges

As described above, the ISS has been the premier in-space platform where we have learned to develop processes and technologies, made groundbreaking discoveries, and opened the doors to new fields of science. Yet, multiple challenges must be solved to enable the continuation of R&D in space. For example, 42 flights were necessary to achieve the fully assembled 1005 m^3^ of pressurized volume on the ISS^1^. Similarly, the ISS was designed in a way that required external spacewalks to be maintained, and this feature has impacted crew time availability to perform research activities. Ironically, the success of R&D on ISS has generated several other challenges, including limitations in stowage (especially temperature-controlled) and competition for on-orbit facilities. These problems have been exacerbated by the relatively low cadence of flights to bring new payloads, the limited up-mass of visiting vehicles, and the lack of down-mass opportunities. While several vehicles have visited the International Space Station, after the space shuttle retired in 2011, only Russia’s Progress, ESA’s ATV (retired in 2015), JAXA’s HTV (retired in 2020), Northrop Grumman’s Cygnus and SpaceX’s Dragon could carry significant cargo to ISS. But because all but Dragon and Soyuz burn up on re-entry, sample return has been limited to these vehicles. This pace delays investigators access to their science, reduces cadence of research, and results in over-committed ISS stowage and freezer space. Other challenges that need to be solved by the CLD community include the ability to upgrade research facilities as quickly as technology advances, and to provide access to non-ISS partner countries and private industry with fewer restrictions. While the intention was set in the 1980s to develop a space station to galvanize the commercialization of LEO, so far these and other challenges have resulted in slow realization^9^.

Orbital Reef’s modules will take advantage of two key innovations: the larger 7-meter diameter fairing of Blue Origin’s New Glenn rocket, and soft-goods and expandable technologies of Sierra Space’s LIFE^TM^ habitat. The LIFE^TM^ module alone offers about a third of ISS’s pressurized volume. Additionally, Orbital Reef is being designed so that it can be maintained from the inside of the space station, avoiding complex EVA operations and helping focus crew time on R&D, production, and revenue-generation efforts. The primary assembly of Orbital Reef will rely on Extravehicular Robotics (EVR), limiting conventional EVAs to contingencies and training missions. Substantial improvements in robotic technologies, as well as designing for robotic assembly and maintenance, will support this economical and safety-driven approach^8^. This reduction in operational expenses translates into lower cost to do research on Orbital Reef for the scientific community.

To address ISS-era transportation challenges, Orbital Reef will utilize Blue Origin’s New Glenn launch system and Sierra Space’s Cargo and Crew Dream Chasers. Additionally, Orbital Reef will be able to receive other vehicles (e.g., Dragon, Boeing Starliner, Cygnus) with both standard berthing and docking interfaces. Furthermore, as a winged vehicle, Dream Chaser will provide a low-g return path to ensure gravity-sensitive samples (e.g., protein crystals) return safely to Earth, and it may land in any runway that can accommodate a Boeing 737 around the world, providing investigators and companies with quick access to their samples or in-space developed products. In addition, our teammates at Amazon/Amazon Supply Chain are reimagining the art of the possible for space logistics. Our robust access to up- and down-mass, together with a philosophy of moving at the speed of business, will allow prompt updates to on-orbit facilities to stay current with the state of art of the science. Robotics and automation will enhance research activities and optimize crew time, and technologies such as augmented reality will connect space-based researchers to their laboratories on Earth, optimizing collaboration and efficiency of research activities. Given its commercial nature, Orbital Reef will provide access to orbit to both ISS and non-ISS partner countries and the private sector. University and Industry R&D Advisory Councils managed by Arizona State University and MIT, respectively, provide focused user inputs to shape the next generation of facilities and processes needed by researchers in academia, government, and industry.

## Discussion

The 2023 ‘Decadal Survey on Biological and Physical Sciences Research in Space’ for 2023–2032, produced by the National Academies of Sciences, Engineering, and Medicine provides recommendations for “a comprehensive vision and strategy for a decade of transformative science at the frontiers of biological and physical sciences research in space”^10^. This report provides long-term strategic advice to NASA and clarity to researchers on which fields of biological and physical sciences are more likely to be supported by the U.S. federal government. Similarly, this decadal survey offers a roadmap towards future facility needs, which community advocacy can leverage to advance the state-of-the-art for science hardware on Commercial LEO Destinations. In this way and many others, investigators can inform CLD developers of their needs, and work with implementation partners to mature their technologies, as described below.

There are numerous ways for the research community to take action now towards conducting research, technology development, and manufacturing activities on Orbital Reef, well before the planned retirement of ISS in 2030. The Orbital Reef team provides a continuum of microgravity research services to payload customers that currently includes suborbital New Shepard flights and soon will include Dream Chaser orbital flights. Both platforms can be used to de-risk projects and collect proof of concept validation. NASA, the ISS international partner agencies, and the ISS National Laboratory have nearly continuous open calls for researchers seeking to leverage the ISS for technology development and both fundamental and applied R&D—to further space exploration and for terrestrial benefit. There are also related funding opportunities from the National Science Foundation (NSF), National Institutes of Health (NIH), Department of Defense (DoD), and others.

In March of 2023, the LEO Science and Technology Interagency Working Group of the U.S. National Science and Technology Council (NSTC) published an interagency strategy and action plan to enable U.S. Government-wide collaboration and support of public-private partnerships to ensure continuity of access and sustainable LEO research and development activities^11^. The research community should continue to advocate for robust government funding to implement this strategy, through publishing thought leadership papers and editorials, responding to NASA Requests for Information (RFI’s), participating in National Academies’ workshops, and shaping the conversation toward the importance of continuous research access to LEO. The research community can also engage directly with the Orbital Reef team to share requirements for next-generation research-enabling facilities in orbit. The research community can position itself to be ready with preliminary data, collaborations, and ground research completed in time to win planned upcoming awards to transition LEO science from ISS to CLDs.

Orbital Reef’s unique environment in Low Earth Orbit offers long-term microgravity, extreme environmental conditions, and a vantage point from which to study the Earth and space. For researchers and organizations who are pushing the limits of understanding and capability, space is a rich environment to probe for new insights and develop new paths to innovation. By reimagining the art of the possible, Orbital Reef will help us take a key step toward a bold vision of millions of people living and working in space for the benefit of Earth.

### Reporting summary

Further information on research design is available in the Nature Research Reporting Summary linked to this article.

### Supplementary information

Reporting Summary
